# Using PDE5 inhibitors for the prevention and treatment of colorectal cancer

**DOI:** 10.1186/2050-6511-16-S1-A11

**Published:** 2015-09-02

**Authors:** Darren D Browning

**Affiliations:** 1 Department of Biochemistry and Molecular Biology, Georgia Regents University, Augusta GA, USA

## Background

There is an emerging consensus that cGMP has functions in the intestinal epithelium that go beyond the regulation of secretion. Observations in mice that are deficient in cGMP signaling components have revealed an important role in epithelial homeostasis, and such animals exhibit defective barrier function and increased susceptibility to intestinal tumorigenesis. Despite recent clinical success with the GCC-agonist Linzess® (linaclotide) in treating constipation, few studies have examined the effect of these agents in colitis or colon cancer models. In addition, a paucity of work focusing on the effects of cGMP elevating agents in wild type mice have left signaling downstream of cGMP and the therapeutic mechanisms poorly understood.

## Results

Our laboratory has reported that clinically relevant phosphodiesterase 5 (PDE5) inhibitors can produce sustained increases in cGMP levels in the intestinal epithelium. In support of previous work with knockout mice, we further demonstrated that PDE5 inhibitors promote the differentiation of colonic secretory cells, and alter homeostasis by suppressing both apoptosis in the luminal epithelium and proliferation in the crypts. These effects of increased cGMP in the colon mucosa led to the suppression of colitis and tumorigenesis in preclinical disease models. The cGMP-dependent signaling pathways identified as likely contributors to the therapeutic effects of PDE5 inhibitors in the gut include PKG2-dependent activation of DUSP10 and FoxO. These pathways are capable of increasing epithelial resilience to redox stress and suppressing tumor proliferation. Preliminary results indicate that these pathways might also be activated by cGMP in the human colon mucosa, which highlights the potential utility of PDE5 inhibitors for the prevention and treatment of colitis and colon cancer.

## Conclusion

Signaling through cGMP has profound physiological effects in the gut mucosa that can be exploited using GCC-agonists and PDE5 inhibitors. While our understanding of the underlying mechanisms is relatively poor, these agents have significant therapeutic potential for the treatment of gastrointestinal diseases.

